# *Blastocystis* Is Associated with Decrease of Fecal Microbiota Protective Bacteria: Comparative Analysis between Patients with Irritable Bowel Syndrome and Control Subjects

**DOI:** 10.1371/journal.pone.0111868

**Published:** 2014-11-03

**Authors:** Céline Nourrisson, Julien Scanzi, Bruno Pereira, Christina NkoudMongo, Ivan Wawrzyniak, Amandine Cian, Eric Viscogliosi, Valérie Livrelli, Frédéric Delbac, Michel Dapoigny, Philippe Poirier

**Affiliations:** 1 CHU Clermont-Ferrand, Centre de Biologie, Laboratoire de Parasitologie-Mycologie, Hôpital G. Montpied, Clermont-Ferrand, France; 2 Clermont Université, Université Blaise Pascal, Laboratoire Microorganismes: Génome et Environnement, BP 10448, Clermont-Ferrand, France; 3 CNRS, UMR 6023, LMGE, Aubière, France; 4 CHU Clermont-Ferrand, Service de Médecine digestive et hépatobiliaire, Hôpital Estaing, Clermont-Ferrand, France; 5 Clermont Université, Université d'Auvergne, UMR 1107 INSERM, Neuro-Dol, Clermont-Ferrand, France; 6 CHU Clermont-Ferrand, DRCI, ‘Délégation Recherche Clinique et Innovation’, Clermont-Ferrand, France; 7 Université Lille Nord de France, INSERM U1019, CNRS UMR 8204, Centre d'Infection et d'Immunité de Lille (CIIL), Institut Pasteur, Biologie et Diversité des Pathogènes Eucaryotes Emergents, Lille, France; 8 Clermont Université, Université d'Auvergne, Centre de Recherche en Nutrition Humaine Auvergne, M2iSH, ‘Microbes, Intestin, Inflammation et Susceptibilité de l'Hôte UMR INSERM/Université d'Auvergne U1071 USC-INRA 2018, BP 10448, Clermont-Ferrand, France; Oregon State University, United States of America

## Abstract

*Blastocystis* is a protistan parasite living in the digestive tract of many animals, including humans. This highly prevalent intestinal parasite is suspected to be linked to Irritable Bowel Syndrome (IBS), a chronic functional bowel disorder. Here, we first compared the prevalence of *Blastocystis* among 56 IBS patients (40 IBS with constipation (IBS-C), 9 IBS with diarrhea (IBS-D), 4 mixed IBS (IBS-M) and 3 unsubtyped IBS (IBS-U) according to the Rome III criteria) and 56 control (*i.e.* without any diagnosed chronic or acute gastrointestinal disorder) subjects. The highest prevalence of *Blastocystis* spp. was observed in the IBS group, but was only statistically significant in men (36.8% in the IBS group *versus* 4.8% in the control group). We then conducted a meta-analysis including epidemiological studies attempting to determine whether *Blastocystis* carriage could be linked to IBS, and highlighted that IBS patients had a relative risk of 2.34 to be infected by *Blastocystis* when compared to non-IBS subjects. We also looked for *Dientamoeba fragilis*, which is often associated with IBS, and identified this parasite only in some IBS patients (n = 6/56). Several studies provided evidence for a major role of the gut microbiota in the pathophysiology of IBS. Thus, we investigated the possible impact of *Blastocystis* carriage on the enteric bacterial community through quantification of 8 major bacterial groups from the enteric flora. Our data indicated that men with IBS-C had a significant decrease in *Bifidobacterium* sp. when infected by *Blastocystis*. Interestingly, in control subjects (*i.e.* without any gastrointestinal disorder) positive for *Blastocystis*, *Faecalibacterium prausnitzii*, which is known for its anti-inflammatory properties, was significantly decreased in men. Our results support the hypothesis that *Blastocystis* might be linked to the pathophysiology of IBS-C and intestinal flora imbalance.

## Introduction

*Blastocystis* sp. is an anaerobic protistan parasite found in the intestinal tract of humans and various animals, with a widespread distribution and characterized by extensive genetic diversity [1]. At least 17 subtypes (STs) have been described on the basis of the sequence of the gene encoding the 18S rRNA, the ST1 to ST9 being recovered from human stool samples [1], [2]. Its prevalence in humans widely varies between countries according to hygienic conditions and sanitary practices [1]. A recent study indicated that this prevalence can reach 100% in a Senegalese population [3]. However, the clinical relevance of *Blastocystis* sp. remains controversial because most cases of infection are asymptomatic [1]. Interest of the scientific and medical communities in *Blastocystis* increased these last few years since epidemiological surveys highlighted a higher prevalence of this parasite in patients suffering from the Irritable Bowel Syndrome (IBS) compared to healthy populations or to patients suffering from other gastrointestinal disorders [4]. IBS is a functional chronic disorder characterized by abdominal pain, bloating and alteration of bowel habits [5]. Its prevalence ranges from 5% to 24% of people living in industrialized countries [6]. The diagnosis of IBS is based on clinical criteria defined by the Rome III classification [6]. Four symptom-based subgroups of IBS can be distinguished according to predominant bowel habit: IBS-C for patients with constipation, IBS-D for patients with diarrhea, IBS-M for patients with alternating diarrhea and constipation and IBS-U for patients with unsubtyped IBS. It is now well recognized that IBS is a multifactorial disorder and the knowledge regarding mechanisms underlying its pathophysiology is still limited. Dysregulation of the “brain-gut” axis is involved in visceral hypersensitivity and intestinal motility disorders [7]. Parietal abnormalities contribute to symptoms with an increased paracellular permeability and a low-grade inflammation of the colonic mucosa [8], [9]. Modifications of the gut microbiota were also reported in IBS patients with an imbalance of *Firmicutes*/*Bacteroidetes* ratio [10]. This imbalance is likely to have impact on gas and metabolite production such as Short Chain Fatty Acids (SCFA). SCFA have been shown to play a role in gut motility and an increase of gas production may contribute to bloating, which is one of the most frequent symptoms observed in IBS subjects [11]. The gut microbiota also strongly interacts with major biological functions such as immunity in participating to mucosal immune system development [12]. Thus, alteration in the composition and/or function of the gut microbiota could have an impact on gut homeostasis. One hypothesis for a mechanism of IBS pathophysiology is that dysbiosis may induce an activation of the mucosal associated innate immune system [13]. Immune imbalance could result in an increase of epithelial permeability associated with an activation of the nociceptive pathways. These cellular disturbances could then contribute to the enteric nervous system dysregulations. In a recent study, Crouzet *et al.* demonstrated that germ free rats inoculated with the fecal microbiota from IBS patients developed visceral hypersensitivity [14]. While the gut microbiota is highly variable between individuals [15], most of the studies reported an increase of *Enterobacteriaceae* in IBS patients associated with a decrease of Lactobacilli and *Faecalibacterium prausnitzii* [16], [17]. *F. prausnitzii* and the subgroup *Clostridium coccoides* are known to produce anti-inflammatory SCFA, in particular butyrate [18]. Butyrate was also shown to improve tight junctions assembly leading to a reduction in paracellular permeability [19].

Until now, only 6 epidemiological studies highlighted a higher prevalence of *Blastocystis* among patients suffering from IBS [20]–[25]. In 5 other surveys, the difference in prevalence between IBS and control subjects was not significant [26]–[30]. In addition, most were carried out in patients from Middle-East, Asia or South-America.

*Dientamoeba fragilis* is another protozoa which is suspected to play a role in the etiology of IBS [23]. Like *Blastocystis*, *D. fragilis* is still neglected because of a high number of asymptomatic carriers and the lack of fulfilled Koch's postulates [31]. Whereas Yakoob *et al.* observed a higher prevalence of *D. fragilis* in IBS-D patients compared to subjects with other gastrointestinal disorders [23], Jimenez-Gonzalez *et al.* highlighted that *D. fragilis* was more frequently found in patients without any history of chronic intestinal disorder [25]. In addition, Engsbro *et al.* showed that eradication of *D. fragilis* was not correlated to an improvement of IBS symptoms [32].

It has been previously reported that both protective and pathogenic bacteria may modulate the proliferation and/or pathogenicity of protozoa such as *Giardia intestinalis* or *Entamoeba histolytica* [33], [34]. A role of the microbiota composition in the high prevalence of *Blastocystis* infection in IBS patients was also recently hypothesized [4], [35].

In our work, we first aimed to evaluate the prevalence of both *Blastocystis* and *D. fragilis* in an IBS population from a westernized country. A meta-analysis including all previous studies reporting prevalence of *Blastocystis* in IBS patients was also performed. In a second part we quantified 8 major bacterial groups from the enteric flora to identify bacterial changes associated to the presence of *Blastocystis*.

## Results

### IBS and control cohorts

56 patients fulfilling Rome III criteria for IBS were recruited (see details in Table 1). Most of them were classified as IBS-C (71.4%), the other were IBS-D (16.1%), IBS-M (7.1%) and IBS-U (5.4%). The control group also included 56 subjects without any history of chronic or acute digestive disease.

**Table 1:** Demographic and epidemiological characteristics of IBS and control groups.

			IBS group				
		Control group	Overall	IBS-C	IBS-D	IBS-M	IBS-U
**Populations**							
**Number**		**56**	**56**	**40**	**9**	**4**	**3**
Male		21	19	12	4	1	2
Female		35	37	28	5	3	1
**Age, years (mean±SD)**		**53.3±16.3**	**53.6±16.0**	**55.1±16.4**	**50.2±16.2**	**45.0±12.0**	**55.3±15.6**
***Blastocystis*** **-positive**							
**Number/total (** ***p*** **-value)**		**9/56**	**13/56(** ***p*** ** = 0.476)**	**11/40(** ***p*** ** = 0.583)**	**1/9**	**1/4**	**0/3**
Male/Total (*p*-value)		1/21	7/19 (*p* = 0.017)	5/12 (*p* = 0.037)	1/4	1/1	n.a.
Female/Total (*p*-value)		8/35	6/37 (*p* = 0.559)	6/28 (*p* = 0.745)	0/4	0/3	n.a.
**Age, years (mean±SD)**		**55.3±19.8**	**52.5±16.4**	**51.6±17.7**	**62**	**52**	**n.a.**
**Subtype distribution**	ST1	0	1	1	0	0	n.a.
	ST2	0	2	2	0	0	n.a.
	ST3	0	3	3	0	0	n.a.
	ST4	8	6	4	1	1	n.a.
	Co-infection	1 [ST2+ST4]	1 [ST2+ST5]	1 [ST2+ST5]	0	0	n.a.
***Dientamoeba fragilis*** **-positive**							
**Number/total (** ***p*** **-value)**		**0/56**	**6/56 (** ***p*** ** = 0.027)**	**4/40**	**1/9**	**1/4**	**0/3**
**Age, years (mean±SD)**		**n.a.**	**55.8±16.4**	**58.3±19.0**	**61**	**41**	**n.a.**

Overall: males plus females, S.D.: standard deviation, n.a.: not applicable, ST: subtype. *P*-values of 0.05 or below were considered as significant (two-sided).

### Prevalence of *Blastocystis* sp

**Figure 1:**
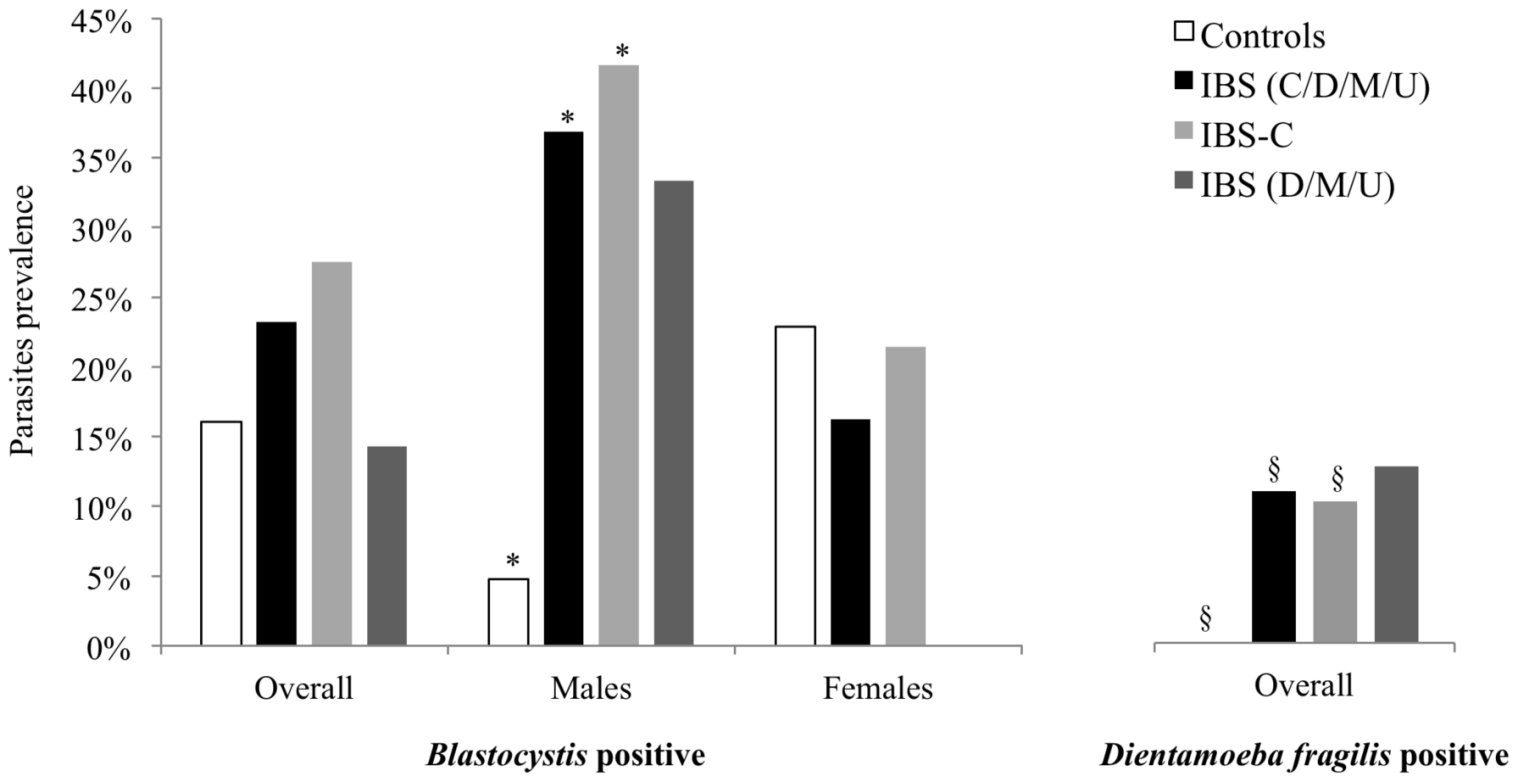
Prevalence of *Blastocystis* and *Dientamoeba fragilis* in both control and IBS groups. Overall: males plus females; *, §: *p*-value p<0.05. The *p*-values are determined by reference to control group.

*Blastocystis* was detected by qPCR in 23.2% (13/56) of patients with IBS and 16.1% (9/56) of control subjects (Table 1 and Figure 1). Interestingly, when considering only males, the prevalence of the parasite in the IBS group (36.8%, 7/19) was significantly higher (*p* = 0.017) than in the control group (4.8%, 1/21). In contrast, the prevalence of *Blastocystis* sp. in females did not significantly differ between both groups (*p* = 0.559). Similar results were observed when considering only the IBS-C subgroup (see Table 1 and Figure 1).

### Prevalence of *Dientamoeba fragilis*

We found that all the 56 control subjects were negative for *D. fragilis* whereas 6 patients (4 women and 2 men) with IBS were positive for the parasite (Figure 1 and Table 1). Three of these patients were also co-infected with *Blastocystis*. However, the number of patients was too low to calculate *p*-values according to subject sex.

### *Blastocystis* subtyping

In the control group, all the 9 *Blastocystis* positive subjects were infected by ST4, including one that was co-infected with ST2 (Table 1). ST4 was also the predominant ST found in *Blastocystis*-positive IBS patients (6/13, Table 1). Among the 7 other patients with IBS, 1 was infected by ST1, 2 by ST2, 3 by ST3 and 1 was co-infected by both ST2 and ST5. However, statistical analysis revealed that the distribution of *Blastocystis* subtypes did not significantly differ between IBS and control groups.

### Meta-analysis of *Blastocystis* prevalence in IBS patients

**Figure 2:**
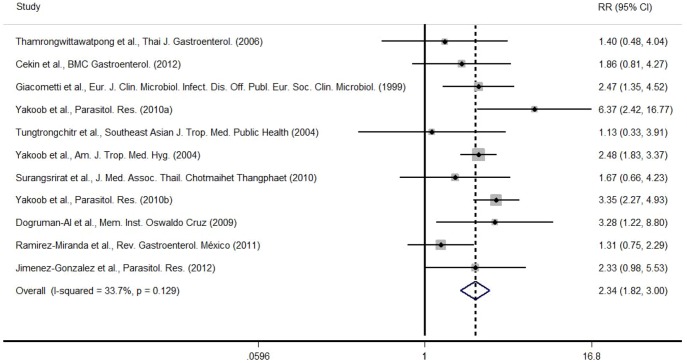
Forest plot of relative risk and 95% confidence interval for *Blastocystis* carriage in IBS subjects. The horizontal lines represent the 95% confidence interval (CI) of the relative risk (RR) for IBS subjects compared to non-IBS subjects in each study. The black box in the middle of the CI represents the single best estimate of RR in that study. The width of the CI is related to the power of the study and inversely associated with sample size. In addition, the pooled or combined RR results of the meta-analysis are represented by a diamond, the width of which is the CI for the pooled data. The vertical line is typically displayed to indicate no effect when RR = 1. When the CI crosses the vertical line of no effect, we must accept the null hypothesis of no difference between two groups. Only if the CI remains clear of the vertical line of no effect can we reject the null hypothesis. In our study, the RR for IBS subjects to carry *Blastocystis* was 2.336 (*p* = 0.001).

A systematic review and meta-analysis was conducted of the 11 published studies to assess the prevalence of *Blastocystis* in patients with IBS. This meta-analysis included 1728 IBS patients and 1292 control subjects (Table S1). IBS patients had a relative risk of 2.336 (*p* = 0.001) to be infected by *Blastocystis* when compared to non-IBS subjects (Figure 2). However, we were unable to perform this analysis when considering only males or females because of numerous missing data in the selected studies.

### Quantification of major bacterial groups from the enteric flora

**Figure 3:**
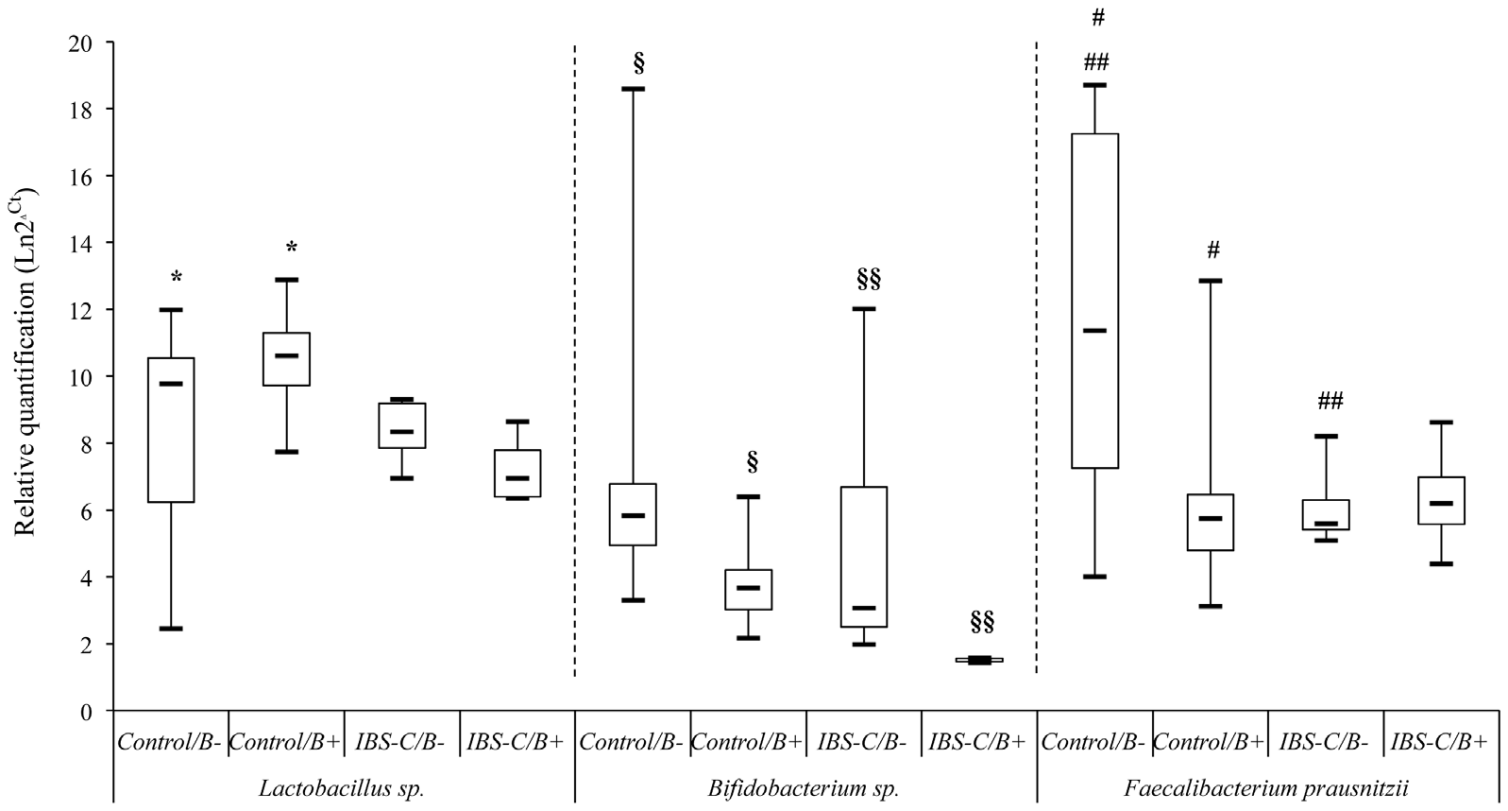
Relative quantification of 3 bacterial groups in IBS-C and control males, according to *Blastocystis* carriage. Lactobacilli, Bifidobacteria and *Faecalibacterium prausnitzii* are quantified by the method of the ΔΔCt in both IBS-C and control males, positive or negative for *Blastocystis*. Boxplots: median (horizontal line in boxes), interquartile range (boxes), max and min values (whiskers). B-: *Blastocystis*-negative; B+: *Blastocystis*-positive; *, §, §§, #, ##: *p*-value p<0.01.

In order to compare the 8 major bacterial communities between IBS-C and control groups, only *Blastocystis*-negative subjects were first considered. *Bacteroides* sp. were significantly increased in patients with IBS-C (*p*<0.001) compared with control group, whereas *Bifidobacterium* sp. (*p*<0.001), *Desulfovibrio* sp. (*p* = 0.011), *C. leptum* (*p* = 0.001) and *F. prausnitzii* (*p* = 0.004) were significantly decreased (Table S2). Statistical analyses after a clustering per sex revealed that *Bacteroides* sp. significantly increased in males (*p* = 0.046) and females (*p* = 0.001), whereas *F. prausnitzii* significantly decreased only in males (*p* = 0.008, Figure 3, Table S2) and both *Bifidobacterium* sp. and *C. leptum* decreased only in females (*p*<0.001 and *p* = 0.004 respectively). When considering *Blastocystis*-positive IBS-C patients, only *Bifidobacterium* sp. were significantly decreased in males (*p* = 0.008, Figure 3, Table S2). No difference was observed for the other bacterial groups.

In the overall control group (*i.e.* without any gastrointestinal disorder), no significant difference between both *Blastocystis*-positive and *Blastocystis*-negative patients was observed (Table S2). However, 3 bacterial groups were shown to be significantly modified in males (Figure 3). Indeed, a significant increase in Lactobacilli was observed in *Blastocystis*-positive males (*p* = 0.043) whereas *Bifidobacterium* sp. (*p*<0.001) and *F. prausnitzii* (*p* = 0.002) were significantly decreased (Figure 3).

## Discussion

Various human diseases are associated with an imbalance of bacteria in the gut, termed dysbiosis, and studies reporting changes in microbiota composition among subgroups of patients with IBS are increasing [10], [16], [17]. Also, intestinal eukaryotic parasites such as *Blastocystis* or *Dientamoeba fragilis* have been shown to be more prevalent in patients suffering from IBS [20]–[25]. However, the role of these parasites in the pathophysiology of IBS is still debated [1], [31]. In addition, most of the studies exploring the prevalence of both *Blastocystis* and *D. fragilis* in IBS cohorts were conducted in Middle-East, Asia or South-America. Because data from westernized countries were missing, we conducted a prospective study in a French cohort. We recruited 56 IBS patients fulfilling Rome III criteria and 56 control subjects and carried out the detection/quantification of both *Blastocystis* and *D. fragilis* by qPCR [36], [37]. Although a higher prevalence of *Blastocystis* was found in IBS patients (23.2% *versus* 16.1% in the control group), this difference was not statistically significant (Figure 1). We then performed a systematic review of the literature and a meta-analysis including previous epidemiological studies in IBS cohorts. Results from this meta-analysis revealed that infection by *Blastocystis* occurs 2.34 times more frequently in patients with IBS than in non-IBS subjects (*p*<0.01, Figure 2). When we compared the prevalence of *Blastocystis* within male and female subgroups (with or without IBS), we observed that the difference was only significant in the males (36.8% for IBS males *versus* 4.8% for control males, Figure 1). This result was confirmed by the multivariate analysis. Among the 56 IBS patients, 40 were IBS-C, 9 IBS-D, 4 IBS-M and 3 unclassified-IBS (Table 1). IBS subgroup is an important parameter to take into account when considering parasite prevalence, as it is thought that causes underlying this syndrome could be different as symptoms appear heterogeneous. Using conventional PCR, Yakoob *et al.* previously reported a higher prevalence of *Blastocystis* in an IBS-D cohort from Pakistan (44% *versus* 21% in controls, *p<0.001*) [23]. In our study, we did not observe any difference in *Blastocystis* carriage between controls and IBS-D/M/U (Figure 1), but the number of patients in these subgroups was too low (Table 1). Since most patients with IBS were subtyped as IBS-C (71.4%), we decided to focus our attention on the prevalence of *Blastocystis* within this subgroup. Here again only significant differences in the prevalence of *Blastocystis* were observed when comparing IBS-C males and control males (41.7% *versus* 4.8%, respectively, see Figure 1). However, because the detailed descriptions of the cohorts were often lacking in other published studies, the meta-analysis did not confirm this difference in *Blastocystis* prevalence between men and women suffering from IBS. Thus, we hypothesized that men could be more exposed to the parasite than women (*via* an environmental reservoir, work, different eating habits …). Nevertheless, we cannot exclude that men suffering from IBS could be more susceptible to the infection by *Blastocystis* since this difference was not observed in the control group. This result is intriguing as women are twice as likely to suffer from IBS than men [38]. This gender disparity is not well understood but likely implies physical, hormonal, social and emotional differences [39]. Thus, the discrepancy between IBS sex-ratio and the distribution of *Blastocystis* in our study remains to be determined.

At least 17 subtypes of *Blastocystis* have been identified, ST1 to ST9 being recovered from human stools with ST3 as the predominant human ST [1]. Some studies suggested the existence of more virulent STs, including ST1 and ST4 [23], [40]. Interestingly, a consistent link between ST1 and IBS-D was identified in a cohort from Pakistan [23]. However, there is no clear evidence between one *Blastocystis* ST and pathogenicity. Further, in our study, the multivariate analysis did not show any association between ST and IBS. Nevertheless, ST4 appeared to be the most prevalent ST in our study (59.1%, Table 1). This high prevalence of ST4 was comparable to that observed (63%) in a previous study we conducted among patients with hematological malignancies [36].

Like *Blastocystis*, the clinical significance of the intestinal protozoa *D. fragilis* remains uncertain as (i) it is commonly isolated from both symptomatic and asymptomatic individuals [31], and (ii) it is often associated with IBS [23]. In our study, *D. fragilis* was only detected in some patients with IBS (n = 6, Figure 1). Three of the six *D. fragilis* positive patients were also infected by *Blastocystis*, confirming the frequently reported occurrence of *D. fragilis* and fecal-oral transmitted parasites co-infections [1], [41].

Gut microbiota modifications reported from IBS subjects primarily associated an increase of *Enterobacteriaceae* with a decrease of both Lactobacilli and Bifidobacteria [10], [16], [17]. While no standard pattern has been associated to one subgroup of IBS, it was shown that fecal flora composition in patients with IBS-D significantly diverged from controls but also from other IBS subgroups [42]. Consequently, we focused our analysis on quantification of the gut microbiota in the IBS-C subgroup as it represents the majority of our IBS patients (71.4%). To strengthen statistical analyses, 13 additional control patients positive for *Blastocystis* were added to the control group and all *D. fragilis* positive patients were excluded. Eight major bacterial groups as representative of the enteric flora, *Enterobacteriaceae*, *Lactobacillus* sp., *Bacteroides* sp., *Bifidobacterium* sp., *Desulfovibrio* sp., *Clostridium coccoides*, *Clostridium leptum* and *Faecalibacterium prausnitzii* were quantified by qPCR (Table S2). We first considered *Blastocystis*-negative subjects in both IBS-C and control groups. Our results in IBS-C patients were consistent with those of other studies [43] including a significant increase of *Bacteroides* sp. While the *Enterobacteriaceae* were increased in patients with IBS-C, the difference with control subjects was not statistically significant (Table S2). Bifidobacteria, *C. leptum* group as well as *F. prausnitzii* were shown to significantly decrease in IBS-C *Blastocystis*-negative subjects. Chassard *et al.* recently reported a significant increase of *Desulfovibrio* in IBS-C patients using a FISH method [44]. *Desulfovibrio* sp. belongs to the sulphate reducing bacteria (SRB) producing H_2_S. H_2_S is known to have various biological effects, and its role in gut homeostasis is still debated [11]. In contrast, we observed that *Desulfovibrio* sp. was significantly decreased in our IBS-C patients. We hypothesized that this discrepancy may be the result of the different methods used for bacterial quantifications. Indeed, discordances in the detection of Bifidobacteria have been reported whether using qPCR or fluorescent in situ hybridization [43].

The presence of *Blastocystis* in both IBS-C and control males was associated with a decrease in Bifidobacteria (Figure 3). *F. prausnitzii* was also significantly reduced in *Blastocystis*-positive males within the control group (Figure 3). As mentioned previously, *F. prausnitzii* was significantly decreased in IBS-C *Blastocystis*-negative subjects. However, we did not observe a supplementary decrease in presence of *Blastocystis*. We supposed that *F. prausnitzii* was probably already strongly decreased in the context of IBS thus preventing to see a significant difference between *Blastocystis*-positive and *Blastocystis*-negative IBS patients. *F. prausnitzii* belongs to the group of *C. leptum* and represents about 5% of the fecal microbiota [45]. A decrease of *F. prausnitzii* was reported from gastrointestinal disorders such as inflammatory bowel diseases (IBD) and IBS. *F. prausnitzii* is considered as an indicator of intestinal health since *in vitro* and *in vivo* assays demonstrated anti-inflammatory effects of this bacteria [18]. Bifidobacteria are widely used as probiotics for their protective effect due to anti-carcinogenic and immunostimulatory properties [46]. Then, both *F. prausnitzii* and Bifidobacteria are considered as protective bacteria. Consumption of Bifidobacteria (as probiotic) could induce in return an increase of *F. prausnitzii* suggesting a putative cross feeding between these bacteria [47]. Their decrease in males in the presence of *Blastocystis* is rather intriguing and suggests that *Blastocystis* carriage could be associated with inflammatory environment. One of the key roles of the gut microbiota is to protect against pathogens. We can assume that the modifications we reported may have favoured the establishment of *Blastocystis*. We recently summarized the hypotheses supporting the association between *Blastocystis* and IBS [4]. Since genomic data from *Blastocystis* ST7 reported the presence of genes encoding a polyketide synthase (PKS) and 2 non ribosomal peptide synthase (NRPS), we hypothesized that *Blastocystis* may have an impact on gut microbiota [4], [48]. Indeed, PKS and NRPS are known to produce a variety of highly effective molecules such as antibiotics [49]. Thus *Blastocystis* may potentially interact with gut microbiota and induce changes in its composition. In their work, Verma *et al.* also reported a depletion of major microbiota genera after infection by *Entamoeba histolytica* [50]. In that field, animal models may probably provide crucial informations. Indeed, the use of gnotobiotic animals would enable to evaluate the direct impact of *Blastocystis* on microbiota composition, or favouring effect of altered microbiota on the establishment of *Blastocystis*.

In summary, we confirmed in a meta-analysis that *Blastocystis* is two times more prevalent in IBS patients than in non-IBS subjects. In our study, this concern was only true in males and associated to significant changes in gut microbiota composition with a decrease of some protective bacteria in IBS-C patients. Nevertheless, our results are self-limited by the method we used. Thus, next generation sequencing tools could be more appropriate to explore whole gut microbiota changes associated to the presence of *Blastocystis*. Even if we cannot conclude whether observed changes in the gut flora were a cause or a consequence of the high prevalence of *Blastocystis* in IBS patients, our results suggest that *Blastocystis* may be used as an indicator of microbiota changes.

## Material and Methods

### Patients and stool samples

Stool samples from 56 patients suffering from IBS were prospectively collected during medical consultation in the Gastroenterology unit at the teaching hospital of Clermont-Ferrand (France) from January 2012 to July 2013. The clinical study was approved by the research ethics committees of the Clermont-Ferrand Hospital (“*Comité de Protection des Personnes Sud-Est 6*”, France) with the reference number 2014/CE29 and which had decided that informed consent from all subjects was not necessary as experiments did not induce additional constraints for patients. IBS diagnosis and symptom-based subgroups of IBS (IBS-C/D/M/U) were established according to the Rome III classification [6]. The control group included 56 patients living in the same geographical area than the group of patients suffering from IBS, and without any history of chronic intestinal disorder nor acute digestive infection at the time of sampling. The stool samples were prospectively collected during the initial systematic screening for digestive colonization with multidrug-resistant bacterial strains. Stool samples from 13 supplementary *Blastocystis*-positive subjects without any history of chronic intestinal disorder were included in the study for the quantification of bacterial groups in order to increase statistical power. All experiments were performed in accordance with relevant guidelines and regulations.

### DNA extraction

Total DNA from 200 mg of each stool sample was extracted using the DNA stool mini kit (Qiagen) according to the manufacturer's recommendations. DNA extracts were stored at −20°C until PCR or qPCR analyses.

### Detection and subtyping of *Blastocystis* sp

Specific quantitative PCR (qPCR) to detect and subtype *Blastocystis* was carried out using BL18SPPF1/BL18SR2PP primers (Table S3) which target a conserved region of the SSU rRNA gene as previously described [36]. PCR products from each positive sample were purified using the Wizard SV Gel and PCR cleanup system (Promega) and sequenced directly (without cloning) by MWG eurofins (Germany) to identify the subtype. In some samples, sequence chromatogram analysis revealed the presence of double traces, suggesting a co-infection by different *Blastocystis* STs. For these samples, a second PCR was performed to amplify and clone a partial sequence of a rDNA marker from the mitochondria-like organelle genome (MLOsrRNA) (Table S3) as recently described [51]. Briefly, the resulting PCR products were purified, cloned in the pGEM-T easy vector (Promega) and transfected into *E. coli* DH5α. Five clones of each sample were arbitrarily sequenced by GATC Biotech (Germany). Sequences were analyzed using the Basic Local Alignment Search Tool (BLAST; http://blast.ncbi.nlm.nih.gov/).

### Detection of *Dientamoeba fragilis*

Specific qPCR to detect *Dientamoeba fragilis* was performed using DF3/DF4 primers and a TaqMan probe (Table S3) based on the sequence of the SSU rRNA gene as previously described [37].

### Bacterial quantifications in stool samples

Most of the recruited IBS patients were classified as IBS-C (n = 40/56). As number of patients from IBS-D, IBS-M or IBS-U groups was too low to reach acceptable statistical power, we only considered IBS-C patients for the microbial quantifications. Because the aim of the study was to analyse changes in bacterial flora associated with the presence of *Blastocystis*, the 4 IBS-C patients infected with *D. fragilis* were excluded. Then, qPCR experiments for bacterial quantifications were carried out for the 36 remaining IBS-C patients, including 8 patients infected with *Blastocystis*. The control group (*i.e.* without IBS) included the 56 subjects previously described (Table 1) and samples from 13 supplementary *Blastocystis* positive patients without any history of chronic digestive disorder of a French multicentric study (personal communication). Eight bacterial groups (*Enterobacteriaceae*, *Lactobacillus*, *Bacteroides*, *Bifidobacterium*, *Desulfovibrio*, *Clostridium coccoides*, *Clostridium leptum* and *Faecalibacterium prausnitzii*) were quantified by specific qPCR as previously described (see primers and probes in Table S3) [52]–[56]. Total bacteria were also quantified by qPCR by targeting the gene encoding the 16S rRNA. All qPCR reactions were run in duplicate. Means of Ct of 16S rRNA qPCR were used to normalize bacterial quantifications by using the ΔΔCt methods. Results were expressed as Ln(2^ΔCt^) for graphical representations.

### Statistical analyses

All analyses were performed using the Stata statistical software (version 13, StataCorp, College Station, US). Qualitative data (including “sex” and “PCR +/−”) were expressed in numbers and associated frequencies, whereas quantitative data (“age” and bacterial quantifications) were expressed as mean (and associated standard deviation) according to the statistical distribution. Considering the non-normality distribution of several bacterial quantifications, a log-transformation was proposed. Then, comparisons between independent groups (“IBS patients”/“control subjects”) were performed by Chi-squared or Fisher's exact tests for qualitative parameters and by Student's t or Mann-Whitney tests when conditions of the t test were not respected (homoscedasticity studied by the Fisher-Snedecor test and normality by the Shapiro-Wilk test). The multivariate analysis (linear or logistic regression according to statistical distribution of dependent variable) was used to study the interaction “sex”×“IBS group”. P-values of 0.05 or below were considered as significant (two-sided). Due to multiple comparisons, the type-I-error inflation was considered when appropriate. Finally, Comprehensive Meta-analysis (version 2, Biostat Corporation) [57] was used with Stata software to conduct the meta-analytical statistical analysis. Heterogeneity in the study results was evaluated by examining forest plots, confidence intervals and using formal tests for homogeneity based on the I^2^ statistics. Random effects meta-analyses were conducted when data could be pooled. Forest plot graph was presented.

## Supporting Information

Table S1 **Meta-analysis of prevalence of** ***Blastocystis*** **in both IBS and control subjects.**
(DOCX)

Table S2 **Quantification of bacterial groups.**
(DOCX)

Table S3 **Primer and probe sequences used in qPCR assays.**
(DOCX)
