# Supplementary material for: Blastocystis Is Associated with Decrease of Fecal Microbiota Protective Bacteria: Comparative Analysis between Patients with Irritable Bowel Syndrome and Control Subjects
Source: PLoS One. 2014 Nov 3;9(11):e111868. doi: 10.1371/journal.pone.0111868 (PMC4218853; doi:10.1371/journal.pone.0111868)
Supplement: Table S1 — Meta-analysis of prevalence of Blastocystis in both IBS and control subjects. (DOCX) [file pone.0111868.s001.docx]

**Table S1. Meta-analysis of prevalence of *Blastocystis* in both IBS and control subjects.** The meta-analysis includes 11 studies published on the topic.

| **Study** | **Prevalence of *Blastocystis* spp.**  **(*Blastocystis* spp. positive subjects/ total number of subjects)** | | |  |
| --- | --- | --- | --- | --- |
|  | **IBS group** | **Control group** | | ***p-value*** |
|  |  |  | Description |  |
| 20. Giacometti *et al.* (1999) Eur J Clin Microbiol Infect Dis Off Publ Eur Soc Clin Microbiol. | 15/81 | 23/307 | “[…] individuals with gastrointestinal disorders other than IBS” | **0.006** |
| 21. Yakoob *et al.* (2004) Am J Trop Med Hyg. | 44/95 | 4/55 | “In control group, there was a recent onset of diarrhea, loss of appetite, fever, and abdominal discomfort.” | **<0.001** |
| 26. Tungtrongchitr *et al.* (2004) Southeast Asian J Trop Med Public Health. | 8/59 | 3/25 | “The study was performed […] with 25 normal subjects serving as controls. Thai volunteers who attended the Out-patient Department-General Practice Sections […], for a physical check-up, were investigated.” | 0.870 |
| 27. Thamrongwittawatpong *et al.* (2006) Thai J Gastroenterol. | 7/40 | 5/40 | “IBS patients, defined by Rome II criteria, who attended the gastroenterology clinic, were registered. Forty patients were enrolled in the IBS group and 40 patients were enrolled in the control group.” | 0.750 |
| 24. Dogruman-Al *et al.* (2009) Mem Inst Oswaldo Cruz. | 8/21 | 5/43 | “While this study did not include a healthy control group, prior study performed at this site with Lugol’s staining has identified the prevalence of Blastocystis infection in healthy controls at 11.6% (5/ 43)” | **0.021** |
| 22. Yakoob *et al.* (2010a) Parasitol Res. | 95/158 | 38/157 | “In control group, there were healthy volunteers or those with upper abdominal discomfort not suggestive of IBS.” | **<0.001** |
| 23. Yakoob *et al.* (2010b) Parasitol Res. | 90/171 | 25/159 | “[…] controls who attended the gastroenterology outpatient clinic” | **<0.001** |
| 28. Surangsrirat *et al.* (2010) J Med Assoc Thail Chotmaihet Thangphaet. | 11/66 | 6/60 | “The control and IBS group were those who attended the gastroenterology clinic during 2007-2008. Those who were not diagnosed of IBS were the control group.” | 0.203 |
| 29. Ramírez-Miranda *et al.* (2011) Rev Gastroenterol México. | 18/115 | 25/209 | “The control group consisted of individuals without diagnosis of IBS but with other physiological alterations and gastrointestinal symptoms i.e., polypus, diabetes, haemorrhoids, ulcerative colitis, etc.” | 0.053 |
| 25. Jimenez-Gonzalez *et al.* (2012) Parasitol Res. | 14/45 | 6/45 | “The control group consisted of individuals with bowel alterations, i.e. polyps, diverticular disease and haemorrhoids, but not with IBS.” | **0.043** |
| 30. Cekin *et al.* (2012) BMC Gastroenterol. | 51/877 | 6/192 | “[…] a control group was selected from population attending the outpatient department within the similar time period [than the study group] on an outpatient basis for routine control without any accompanying gastrointestinal symptoms. Patients in the control group were not diagnosed with any gastrointestinal diseases (IBD, IBS etc.) and, they were also checked for fecal occult blood.” | 0.133 |
| **Total** | **361/1728** | **146/1292** |  | / |
