# Supplementary material for: Blastocystis Is Associated with Decrease of Fecal Microbiota Protective Bacteria: Comparative Analysis between Patients with Irritable Bowel Syndrome and Control Subjects
Source: PLoS One. 2014 Nov 3;9(11):e111868. doi: 10.1371/journal.pone.0111868 (PMC4218853; doi:10.1371/journal.pone.0111868)
Supplement: Table S2 — Quantification of bacterial groups. (DOCX) [file pone.0111868.s002.docx]

**Table S2. Quantification of bacterial groups.**

| Group / *Blastocystis* qPCR result | Subjects | *Enterobacteriaceae* | | | | | *Lactobacillus* sp. | | | | | *Bacteroïdes* sp. | | | | | *Bifidobacterium* sp. | | | |
| --- | --- | --- | --- | --- | --- | --- | --- | --- | --- | --- | --- | --- | --- | --- | --- | --- | --- | --- | --- | --- |
|  |  | **Mean** | **Sd** | | ***p*-value** | | **Mean** | **Sd** | | ***p*-value** | | **Mean** | **Sd** | | ***p*-value** | | **Mean** | **Sd** | ***p*-value** | |
| IBS-C / *Blastocystis* - | All | 6.625 | 3.106 | | 0.147 | | 7.612 | 1.268 | | 0.061 | | 4.907 | 0.824 | | **<0.001** | | 2.915 | 2.842 | **<0.001** | |
| Control group/ *Blastocystis* - |  | 5.686 | 3.747 | |  |  | 7.902 | 3.526 | |  |  | 3.909 | 3.925 | |  |  | 5.956 | 3.871 |  |  |
| IBS-C / *Blastocystis* - | Females | 6.166 | 3.109 | | 0.071 | | 7.354 | 1.283 | | 0.069 | | 4.897 | 0.846 | | **0.001** | | 2.202 | 2.143 | **<0.001** | |
| Control group/ *Blastocystis* - |  | 4.797 | 3.458 | |  |  | 7.657 | 3.850 | |  |  | 3.535 | 4.280 | |  |  | 5.099 | 3.700 |  |  |
| IBS-C / *Blastocystis* - | Males | 8.002 | 2.870 | | 0.439 | | 8.384 | 0.906 | | 0.507 | | 4.938 | 0.815 | | **0.046** | | 5.054 | 3.733 | 0.086 | |
| Control group/ *Blastocystis* - |  | 6.885 | 3.873 | |  |  | 8.233 | 3.100 | |  |  | 4.414 | 3.430 | |  |  | 7.113 | 3.886 |  |  |
| IBS-C / *Blastocystis* - | All | 6.625 | 3.106 | | 0.209 | | 7.612 | 1.268 | | 0.518 | | 4.907 | 0.824 | | 0.094 | | 2.915 | 2.842 | 0.304 | |
| IBS-C / *Blastocystis* + |  | 5.310 | 1.823 | |  |  | 8.054 | 1.666 | |  |  | 5.668 | 1.234 | |  |  | 2.002 | 1.088 |  |  |
| IBS-C / *Blastocystis* - | Females | 6.166 | 3.109 | | 0.824 | | 7.354 | 1.283 | | 0.075 | | 4.897 | 0.846 | | 0.159 | | 2.202 | 2.143 | 0.824 | |
| IBS-C / *Blastocystis* + |  | 5.855 | 2.328 | |  |  | 8.884 | 1.861 | |  |  | 5.809 | 1.388 | |  |  | 2.498 | 1.449 |  |  |
| IBS-C / *Blastocystis* - | Males | 8.002 | 2.870 | | 0.059 | | 8.384 | 0.906 | | 0.089 | | 4.938 | 0.815 | | 0.345 | | 5.054 | 3.733 | **0.008** | |
| IBS-C / *Blastocystis* + |  | 4.765 | 1.241 | |  |  | 7.224 | 1.084 | |  |  | 5.526 | 1.255 | |  |  | 1.510 | 0.074 |  |  |
| Control / *Blastocystis* + | All | 6.174 | 4.222 | | 0.787 | | 8.989 | 2.334 | | 0.341 | | 3.839 | 1.828 | | 0.558 | | 4.287 | 1.570 | **<0.001** | |
| Control / *Blastocystis* - |  | 5.686 | 3.747 | |  |  | 7.902 | 3.526 | |  |  | 3.909 | 3.925 | |  |  | 5.956 | 3.871 |  |  |
| Control / *Blastocystis* + | Females | 4.729 | 2.605 | | 0.946 | | 7.239 | 2.034 | | 0.151 | | 4.214 | 2.206 | | 0.347 | | 4.898 | 1.866 | 0.338 | |
| Control / *Blastocystis* - |  | 4.797 | 3.458 | |  |  | 7.657 | 3.850 | |  |  | 3.535 | 4.280 | |  |  | 5.099 | 3.700 |  |  |
| Control / *Blastocystis* + | Males | 7.378 | 4.999 | | 0.785 | | 10.446 | 1.385 | | **0.043** | | 3.527 | 1.472 | | 0.969 | | 3.777 | 1.110 | **<0.001** | |
| Control / *Blastocystis* - |  | 6.885 | 3.873 | |  |  | 8.233 | 3.100 | |  |  | 4.414 | 3.430 | |  |  | 7.113 | 3.886 |  |  |
| Group / *Blastocystis* qPCR result | **Subjects** | ***Desulfovibrio* sp.** | | | | | ***C. coccoides*** | | | | | ***C. leptum*** | | | | | ***F. prausnitzii*** | | | |
|  |  | **Mean** | | **Sd** | | ***p*-value** | **Mean** | | **Sd** | | ***p*-value** | **Mean** | | **Sd** | | ***p*-value** | **Mean** | **Sd** | | ***p*-value** |
| IBS-C / *Blastocystis* - | All | 6.880 | | 1.358 | | **0.011** | 6.214 | | 3.900 | | 0.057 | 3.077 | | 4.308 | | **0.001** | 6.809 | 2.952 | | **0.004** |
| Control / *Blastocystis* - |  | 8.414 | | 3.250 | |  | 9.085 | | 5.612 | |  | 6.994 | | 6.505 | |  | 9.960 | 5.711 | |  |
| IBS-C / *Blastocystis* - | Females | 7.092 | | 1.373 | | 0.060 | 5.925 | | 3.853 | | 0.216 | 2.710 | | 3.961 | | **0.004** | 7.066 | 3.339 | | 0.161 |
| Control / *Blastocystis* - |  | 8.543 | | 3.395 | |  | 7.821 | | 4.885 | |  | 6.037 | | 5.887 | |  | 9.037 | 6.017 | |  |
| IBS-C / *Blastocystis* - | Males | 6.243 | | 1.173 | | 0.077 | 7.081 | | 4.219 | | 0.347 | 4.180 | | 5.416 | | 0.086 | 6.039 | 1.068 | | **0.008** |
| Control / *Blastocystis* - |  | 8.240 | | 3.121 | |  | 10.790 | | 6.186 | |  | 8.288 | | 7.208 | |  | 11.205 | 5.153 | |  |
| IBS-C / *Blastocystis* - | All | 6.880 | | 1.358 | | 0.581 | 6.214 | | 3.900 | | 0.246 | 3.077 | | 4.308 | | 0.939 | 6.809 | 2.952 | | 0.361 |
| IBS-C / *Blastocystis* + |  | 6.867 | | 1.562 | |  | 6.782 | | 3.174 | |  | 2.092 | | 1.207 | |  | 7.068 | 1.973 | |  |
| IBS-C / *Blastocystis* - | Females | 7.092 | | 1.373 | | 0.283 | 5.925 | | 3.853 | | 0.182 | 2.710 | | 3.961 | | 0.459 | 7.066 | 3.339 | | 0.266 |
| IBS-C / *Blastocystis* + |  | 6.807 | | 2.228 | |  | 7.870 | | 4.430 | |  | 2.546 | | 1.471 | |  | 7.781 | 2.162 | |  |
| IBS-C / *Blastocystis* - | Males | 6.243 | | 1.173 | | 0.257 | 7.081 | | 4.219 | | 0.706 | 4.180 | | 5.416 | | 0.257 | 6.039 | 1.068 | | 0.706 |
| IBS-C / *Blastocystis* + |  | 6.926 | | 0.845 | |  | 5.693 | | 0.848 | |  | 1.638 | | 0.828 | |  | 6.354 | 1.745 | |  |
| Control / *Blastocystis* + | All | 7.240 | | 2.434 | | 0.102 | 9.689 | | 6.605 | | 0.923 | 7.493 | | 8.095 | | 0.699 | 6.064 | 2.979 | | **0.001** |
| Control / *Blastocystis* - |  | 8.414 | | 3.250 | |  | 9.085 | | 5.612 | |  | 6.994 | | 6.505 | |  | 9.960 | 5.711 | |  |
| Control / *Blastocystis* + | Females | 7.868 | | 2.484 | | 0.432 | 9.073 | | 7.020 | | 0.932 | 7.196 | | 8.168 | | 0.918 | 6.197 | 3.622 | | 0.104 |
| Control / *Blastocystis* - |  | 8.543 | | 3.395 | |  | 7.821 | | 4.885 | |  | 6.037 | | 5.887 | |  | 9.037 | 6.017 | |  |
| Control / *Blastocystis* + | Males | 6.717 | | 2.367 | | 0.120 | 10.201 | | 6.506 | | 0.726 | 7.740 | | 8.389 | | 0.586 | 5.953 | 2.484 | | **0.001** |
| Control / *Blastocystis* - |  | 8.240 | | 3.121 | |  | 10.790 | | 6.186 | |  | 8.288 | | 7.208 | |  | 11.205 | 5.153 | |  |

*Blastocystis* –: *Blastocystis*-negative; *Blastocystis* +: *Blastocystis*-positive; Sd: standard deviation
