# Supplementary material for: Blastocystis Is Associated with Decrease of Fecal Microbiota Protective Bacteria: Comparative Analysis between Patients with Irritable Bowel Syndrome and Control Subjects
Source: PLoS One. 2014 Nov 3;9(11):e111868. doi: 10.1371/journal.pone.0111868 (PMC4218853; doi:10.1371/journal.pone.0111868)
Supplement: Table S3 — Primer and probe sequences used in qPCR assays. (DOCX) [file pone.0111868.s003.docx]

**Table S3. Primer and probe sequences used in qPCR assays.**

| **Target** | **Name** | **Sequence 5’-3’** | **References** |
| --- | --- | --- | --- |
| *Dientamoeba fragilis* | DF3  DF4  DP | GTT GAA TAC GTC CCT GCC CTT T  TGA TCC AAT GAT TTC ACC GAG TCA  FAM-CAC ACC GCC CGT CGC TCC TAC CG-TAMRA | 37 |
| *Blastocystis* spp. (SSU rRNA) | BL18SPPF1  BL18SR2PP | AGT AGT CAT ACG CTC GTC TCA AA  TCT TCG TTA CCC GTT ACT GC | 36 |
| *Blastocystis* spp. (MLOsrRNA) | MLOsrDNA-D1  MLOsrDNA-R1 | GAC ATT GAT AGA CGA AAG  GTA GCA CAT GTG TAG CCC | 51 |
| *16S rRNA* (total bacteria) | F_Bact1369  P_TM1389F | CGG TGA ATA CGT TCC CGG  CTT GTA CAC ACC GCC CGT C | 55 |
| *Enterobacteriaceae* | Eco1457F  Eco1652R | CAT TGA CGT TAC CCG CAG AAG AAG C  CTC TAC GAG ACT CAA GCT TGC | 54 |
| *Bacteroides* sp. | F_Bacter11  R_Bacter08  P_Bac303 | CCT WCG ATG GAT AGG GGT T  CAC GCT ACT TGG CTG GTT CAG  VIC-AAG GTC CCC CAC ATT G | 55 |
| *Lactobacillus* sp. | F_Lacto05  R_Lacto04 | AGC AGT AGG GAA TCT TCC A  CGC CAC TGG TGT CTY TCC ATA TA | 55 |
| *Desulfovibrio* sp. | DSV691-F  DSV826-R | CCG TAG ATA TCT GGA GGA ACA TCA G  ACA TCT AGCATC CAT CGT TTA CAG C | 52 |
| *Bifidobacterium* sp. | LM26  Bif228 | GAT TCT GGC TCA GGA TGA ACGC  CTG ATA GGA CGC GAC CCC AT | 53 |
| *C. coccoides* | F_Ccoc07  R_Ccoc14 | GAC GCC GCG TGA AGG A  AGC CCC AGC CTT TCA CAT C | 55 |
| *C. leptum* | F_Clept 09  R_Clept 08 | CCT TCC GTG CCG SAG TTA  GAA TTA AAC CAC ATA CTC CAC TGC TT | 55 |
| *F. prausnitzii* | Fpra428F  Fpra583R  Fpra493PR | Tgt aaa ctc ctg ttg ttg agg aag ata  Gcg ctc cct tta cac cca  Fam-caa gga agt gac ggc ta acta cgt gcc ag-tamra | 56 |
